# Neurological outcomes after cardiac arrest: cold and dark issues

**DOI:** 10.5935/0103-507X.20150051

**Published:** 2015

**Authors:** Cristina Granja, Antonio Paulo Nassar Junior

**Affiliations:** 1 Department of Biomedical Sciences and Medicine, Universidade do Algarve - Faro, Portugal.; 2 Discipline of Clinical Emergency, Hospital das Clínicas, Faculdade de Medicina, Universidade de São Paulo - São Paulo (SP), Brazil.; 3 Adult Intensive Care Unit, A.C. Camargo Cancer Center - São Paulo (SP), Brazil.

Prognostication after cardiac arrest is important for patients, families and health providers. It also has ethical and social implications. With the introduction of therapeutic hypothermia after recovery from cardiac arrest in comatose patients,^(1,2)^ prognostication has become more complex and concerns have been raised, particularly about the amount of time and the number of tools required for this treatment.

The 2010 guidelines^(3)^ emphasized the lack of high-level studies that support the use of any imaging modality to predict the outcomes of comatose cardiac arrest survivors and supported the view that decisions to limit care should not be made based on the results of a single prognostication tool. Since then, some progress has been made. The 2015 guidelines highlight the need for a careful, daily, clinical neurological examination as the foundation for prognostication and reference the existence of multiple studies that support the use of multiple testing modalities that might be categorized as follows: clinical examination; neurophysiological studies - somatosensory evoked potentials and electroencephalography; biochemical markers - neuron-specific enolase as the most commonly used; imaging studies - brain computed tomography and magnetic resonance imaging (MRI).^(4)^

Emphasis was also placed on the timing of prognostication as the recommendations for prognostication have become clearer. The earliest time to prognosticate a poor neurologic outcome using a clinical examination for patients not treated with targeted temperature management is 72 hours after the return of spontaneous circulation. However, this time can be longer after cardiac arrest if the residual effect of sedation or paralysis is suspected to confound the clinical examination. In patients treated with targeted temperature management, in which sedation or paralysis could confound a clinical examination, waiting 72 hours after the return to normothermia is recommended. An algorithm for the management of post-resuscitation care is suggested, with an emphasis on the use of multimodal prognostication whenever possible.^(4)^

The study by Leão et al., published in this issue of RBTI,^(5)^ presents additional data to use when informing relatives about the neurological outcomes after cardiac arrest. The authors found that hypoxic-ischemic brain injury observed on MRI and neuron-specific enolase were strongly associated with a poor neurological outcome (complete dependency for daily living activities, coma or vegetative state).

Since the study by Nielsen et al.,^(6)^ which suggested that maintaining a targeted normothermia had similar outcomes compared with TH for unconscious survivors of cardiac arrest, interest in performing TH has decreased. The 2015 guidelines recommend targeting a temperature of 32 - 36°C during the first 24 hours for unconscious survivors of cardiac arrest. A previous study randomized patients to cooling prior to hospital admission or standard treatment. The intervention group reached the target temperature approximately 1 hour before the standard group. There was no difference in mortality or neurological status at hospital discharge. Thus, this study previously suggested that an early achievement of TH is not associated with better outcomes.^(7)^ The findings of increased mortality and worse neurological outcomes with an earlier achievement of TH by Leão et al.^(5)^ demonstrate additional reasons to be cautious before implementing TH in cardiac arrest survivors. Although this finding is unexpected and is derived from a small observational study, there are known possible explanations. First, in animal models it is well-known that hypothermia can decrease coronary perfusion, which is associated with worse outcomes.^(8)^ Second, severely neurologically impaired patients are known to have impaired thermoregulatory control and may be less reactive to hypothermia.^(9)^

Therefore, despite the limitations of the study by Leão et al.^(5)^ and of those previously mentioned studies, we think that the main findings and, in particular, the non-beneficial effect of starting TH earlier raises important questions that need to be addressed in future studies. Meanwhile, targeting a lower normothermia seems to be a wise choice. However, hypoxic-brain injuries observed on MRI and high levels of neuron-specific enolase after 24 hours are indicative of poor outcomes.
